# Atlantoaxial Misalignment Causes High Blood Pressure in Rats: A Novel Hypertension Model

**DOI:** 10.1155/2017/5986957

**Published:** 2017-07-16

**Authors:** Zong-Bao He, You-Kui Lv, Hui Li, Qiong Yao, Ke-Ming Wang, Xiao-Ge Song, Zi-Jian Wu, Ximing Qin

**Affiliations:** ^1^Department of Rehabilitation, Anhui People's Armed Police Corps Hospital, Hefei, Anhui 230041, China; ^2^School of Acupuncture and Osteology, Anhui University of Traditional Chinese Medicine, Hefei, Anhui 230038, China; ^3^Institute of Health Sciences, Anhui University, Hefei, Anhui 230601, China

## Abstract

Atlantoaxial disorders are often correlated with hypertension in practice. In order to study the relationship between atlantoaxial disorder and hypertension, we attempted to construct an animal model. In this work, we presented an animal model where their atlantoaxial joints were misaligned. We investigated the changes of blood pressure before and after treatments of the modeled rats. We had the following results. (1) SBP and DBP of each surgery group were significantly higher than those of control and sham groups. (2) After the second operation (the fixture was removed), SBP and DBP of both surgery groups decreased and got closer to the control and sham groups after 7 days. (3) Heart rates got significantly higher in both surgery groups, compared to control and sham groups. (4) The blood Ach levels of the surgery groups were significantly lower than those of control and sham groups. With these results, we concluded that we successfully constructed cervical atlantoaxial disorder models in rats that showed hypertension symptom. However, the underlying mechanism connecting atlantoaxial disorder and hypertension still requires further study.

## 1. Introduction

High correlation was observed between cervicogenic diseases and hypertension [1–6], which has attracted close attention from medical scholars [7–9]. Anatomical abnormalities of the cervical spine at the level of the upper vertebra are associated with increased blood pressure (BP) [10, 11]. Manual correction of this mal-alignment has been associated with reduced arterial pressure [12–15]. The study by Bakris et al. reported that 50 patients with Stage 1 hypertension were recruited to receive a National Upper Cervical Chiropractic procedure or a sham procedure randomly and concluded that restoration of Atlas alignment is associated with marked and sustained reductions in BP [8]. However, the underlying mechanism is not clear. Early studies from us [16, 17] and Sun et al. [18, 19] found that stimulations of rabbit anterior cervical ganglion but not tractions of vertebral artery would induce increasement of blood pressure. Cassaglia et al. [20] reported that the activation of goat superior cervical ganglia (SCG) had increased arterial pressure. Many mammals, including humans, have cerebral blood vessels that can receive either vasoconstrictor or vasodilator nerves. Basilar arterial blood flow was found to be enhanced when cerebral sympathetic nerves were activated [21]. Our previous work in rabbits had demonstrated that activation of SCG can increase blood pressure and level of norepinephrine (NE) and decrease level of acetylcholine (Ach) [16, 17]. Though we gained knowledge that activation of the sympathetic nerves would increase blood pressure, we still lack the direct evidence of how atlantoaxial disorder affects blood pressure. Thus, we sought to construct an animal model of atlantoaxial disorder in order to study the relationship between hypertension and cervical vertebrate disorder.

We present in this study an animal model where their atlantoaxial joints were misaligned by inserting artificial implants (24-6 staples, 6 mm long). We chose staples as implants because they have high developing degrees under X-ray imaging system and they can be fixed in the joints without being loose. Since the blood pressure gets better for these cervicogenic hypertension patients when they received cervical vertebra reduction manipulation therapy [8, 15], we asked whether these modeled animals can relieve their high blood pressure after removing these implants. Atlantoaxial disorders are often accompanied with abnormal autonomic nerve activities [22]. In our patients, we found that rotary of head and neck joint or fixation of the left occipital cervical vertebrae will appear as obvious symptom of blood pressure increasement [15]. We have observed many X-ray images from the patients with the cervicogenic disorder and found their atlantoaxial joints often being moved to the left side. Anatomical and physiological structures of both sides of cervical vertebrae are different [23–26], so we constructed two experimental groups, left group (LG) and right group (RG), to differentiate their impacts.

This study presents a successful cervicogenic hypertension rat model that has never been reported [27], according to our knowledge. The implants-caused hypertension was relieved after removing the inserted fixtures. We also monitored the heart rate and the blood acetylcholine level. We report here close correlations between our hypertension model and these two parameters. This study will not only reveal the mechanism that underlies atlantoaxial disorder which could cause hypertension but also provide experimental basis for our practical manipulation on these cervicogenic hypertension patients.

## 2. Materials and Methods

### 2.1. Experimental Animals

130 SD male rats (six weeks old, weighing 250 ± 10 g, purchased from Anhui Provincial Animal Center) were housed in a temperature of 23–26°C, humidity of 50–60%, and light intensity of controlled environment for one week. They were randomly divided into the control group (*n* = 30) and the sham group (*n* = 30). The remaining 70 rats were prepared for receiving implant fixtures to construct atlantoaxial disorder model animals (see next section). These animals were raised under conditions approved by the Animal Care and Use Committee at Anhui University of Chinese Medicine (light, 0600–1800 h; dark, 1800–0600 h). We chose 70 rats because we had dead animals and the animals that cannot fit our criteria during the surgeries as described below.

### 2.2. Methods of Inserting Implant Fixtures and Removing Fixtures from Rats

To construct the left group (LG) with surgery, SD male rats were anesthetized by 10% chloral hydrate (0.3 ml/100 g) and the atlantoaxial joints were exposed carefully, with the ligaments on the left side being partially cut. The head of anesthetized animals was rotated to left side about 15 degrees, and then sterile implant fixtures (24-6 staple) were inserted into the left atlantoaxial joint. After the surgery, the skin incision was sutured, and the animals were injected with penicillin intraperitoneally (as illustrated in Supplemental Figure 1 in Supplementary Material available online at https://doi.org/10.1155/2017/5986957).

In right group (RG), the surgery process is the same as that of LG but the position was changed to the right. In the sham group, an incision was made on rats' cervical skin and the ligaments were partially cut, and then the incision was sutured.

Finally, we had 30 rats for LG group and 30 rats for RG group. Before and after the surgery, they were selected by inclusion criteria. The inclusion criteria are as the following: the animals have no atlantoaxial joint problem before the surgery, judged by X-ray images; dead animals and these animals that show abnormal behaviors were excluded; the animals that do not show expected atlantoaxial joint change were excluded; and all animals show normal electrocardiogram (Power lab-8, Ad Instruments, Australia).

To remove the implanted fixtures, half of the rats of each surgery group (the other half were used for other measurements such as the blood Ach level in next sections) took a second surgery to remove the inserts, and then the incisions were sutured and penicillin was given to these animals.

### 2.3. Measurements of Blood Pressure and Heart Rate

Blood pressure was recorded at the same time on corresponding days, indicated in the main text, when the animals were in their calm state (Rat Blood Pressure System, IITC Life Science, United States). Heart rates were recorded after the first surgery (Power lab-8, Ad Instruments, Australia).

### 2.4. Measurements of the Blood Ach Level

After the blood pressure was recorded on the last day of the 1st surgery, 4–6 ml blood was drawn from abdominal aorta into anticoagulation tubes, and then the serum was collected from half of the rats of each group. Blood Ach level was measured by the following instructions from ELISA kit (ESK6427-48T, Sangon Biotech, Shanghai). Signals were read with a microplate reader (IMARK, Bio-Rad, United States).

### 2.5. Statistical Analysis

Statistics analyses were performed with SPSS Statics 19.0 program. All data are presented with mean ± s.e. (standard error) and analyzed with one-way ANOVA. Groups were compared using Dunnett's test, and *p* < 0.05 was considered significant. Figures were illustrated by Excel 2003.

## 3. Results

### 3.1. Experimental System to Mimic Atlantoaxial Structure Disorder in Rats

**Figure 1:**
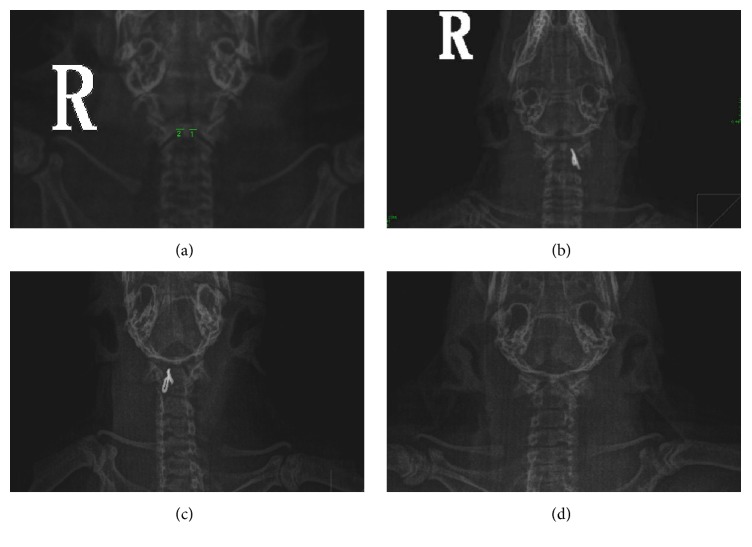
*X-ray imaging to judge atlantoaxial disorder rats*. (a) The atlantoaxial joints of normal rats under X-ray system. (b) The implanted fixture was inserted into the left atlantoaxial joint. (c) The implanted fixture was inserted into the right atlantoaxial joint. (d) X-ray images after the implant was removed.

We chose male SD rats as our experimental model to study whether atlantoaxial structure misalignment/disorder would cause hypertension. Under normal conditions, the cervical spine is balanced by interactions with muscles, ligaments, and bones. We had attempted to break the balance by placing artificial implants into the atlantoaxial joint. The animals were divided into control group, sham group, and the experimental groups which were separated into two groups: left group (LG) and right group (RG). In Supplemental Figure 1, we present the experimental process of making an atlantoaxial disorder in six-week-old rats. The atlantoaxial joints were exposed and the implants were inserted into appropriate positions to LG and RG, as described in the section of* Materials and Methods *accordingly (Figures 1(b) and 1(c)).

In clinic, the standard to evaluate atlantoaxial disorder is by estimating position changes between different joints, including* articulatio atlantoaxialis mediana* and* articulatio atlantoaxialis lateralis*. In order to study the atlantoaxial disorder in lab animals, first we need to develop a method to assess atlantoaxial disorder in rats. X-ray imaging was applied to measure the distances between odontoid and massa lateralis atlantis (see Figure 1(a), distances of both sides are labeled). We had optimized our medical X-ray imaging system since we had found that rats with anterior position were optimal for imaging (see Supplemental Figure 2). From the X-ray images, we had made a standard that can assess rats with atlantoaxial disorder: the distance between odontoid and massa lateralis atlantis is larger than 5 mm, and the joint spaces of both sides are asymmetrical. Control and sham group rats are normal when they were judged by the images: equilibrate distances between odontoid and both sides of massa lateralis atlantis, with difference within 0.01 ± 0.13 mm.

During our surgery, we had achieved 96% and 93% of survival rates for the LG and RG, respectively. Among these survived animals, 26 rats within LG and 24 within RG have appeared with atlantoaxial disorder, evaluated by X-ray images. Our control and sham groups were normal under the X-ray images. After successfully making rats model, we began to monitor the blood pressure with them.

### 3.2. Hypertensive Rats Caused by Atlantoaxial Disorder

**Figure 2:**
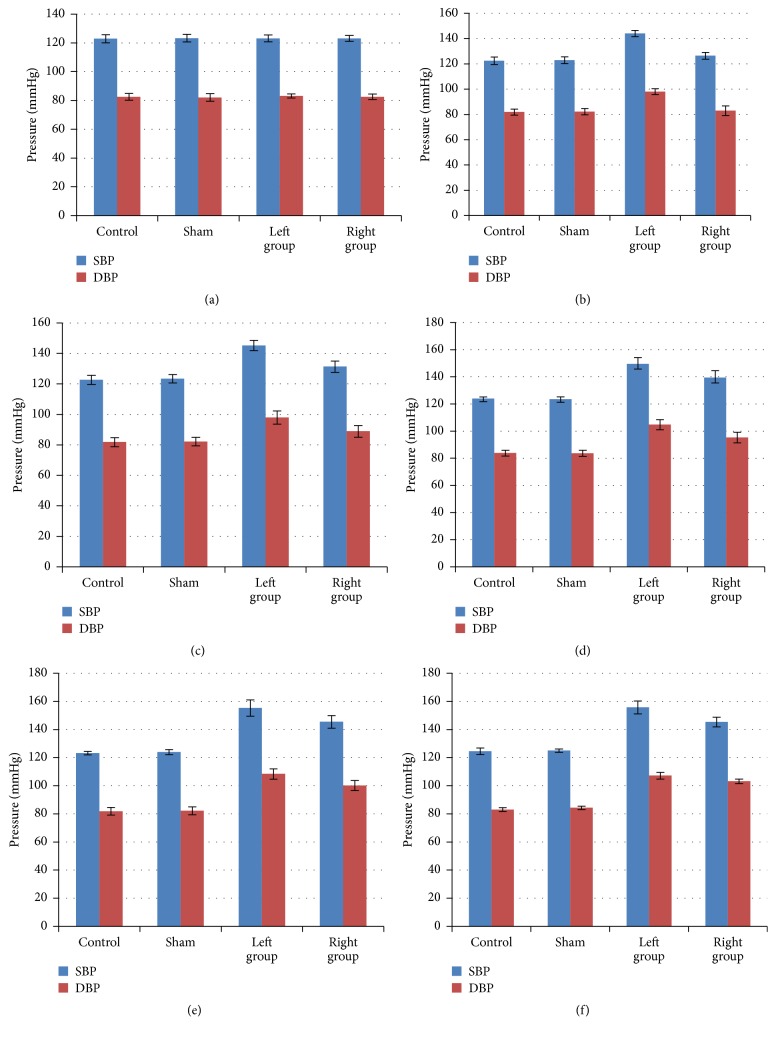
*Blood pressures of atlantoaxial disorder rats increased after the surgery*. (a) Blood pressures were recorded before the surgery and there was no difference between each group with either SBP or DBP (*p* > 0.05, Dunnett's test). After the surgery, the blood pressure was recorded on the first (b), second (c), third (d), fifth (e), and seventh days (f), continuously. Since the first day, the SBP and DBP of both experimental groups (LG and RG) were significantly higher than these of control and sham groups (*p* < 0.05, either LG or RG compared with the sham group). *n* = 30 for each group.

Before surgery, there was no difference of the blood pressures among four groups, including SBP, systolic blood pressure, and DBP (diastolic blood pressure) (Figure 2(a)). After the artificial implants were inserted into the left or right atlantoaxial joints, we monitored the blood pressure changes on the first, second, third, fifth, and seventh days, continuously. As illustrated by Figure 2, compared to the control and sham groups, SBP and DBP of both experimental groups (LG and RG) were gradually increasing (Figures 2(b), 2(c), 2(d), 2(e), and 2(f)). The numbers for SBP were from 123.11 ± 2.40 mmHg to 155.75 ± 4.62 mmHg and 123.16 ± 2.07 mmHg to 145.39 ± 3.47 mmHg, respectively (Supplemental Table 1). Similar trends were seen in DBP. Both groups reached a plateau phase 5 days after the surgery, while SBP and DBP of LG increased significantly faster than those of RG (Supplemental Table 1). We concluded that these experimental rats were considered as hypertension animal models.

### 3.3. Hypertension Changed to Normotension in Experimentally Joint Disorder Relieved Rats

**Figure 3:**
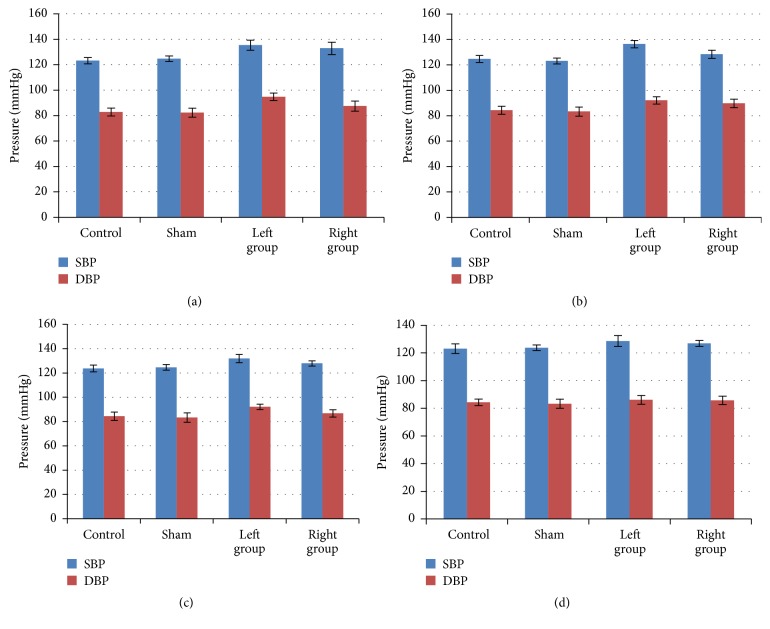
*Blood pressure returned to normal after removing the implanted fixtures*. After removing the implanted fixture, the blood pressure was recorded on the first (a), second (b), fifth (c), and seventh days (d), continuously. The blood pressure level decreased immediately (on the first day) after this surgery and eventually, the SBP and DBP of both experimental groups (LG and RG) were not different from these of control and sham groups (*p* > 0.05, Dunnett's test). *n* = 15 for each group, since half animals were blood drawn.

In clinic, we found that the blood pressure in patients turns better when their cervical spines were restituted after rehabilitation therapy. Then we asked whether the blood pressure changes in our experimental rats would get better if these implanted fixtures were removed (Figure 1(d)). Indeed, we observed an immediate reduction of blood pressures for both SBP and DBP in our rats on the first day after taking out the inserts (Figure 3(a)).

SBP and DBP of both experimental groups (LG and RG) were gradually decreasing (Figures 3(b), 3(c), and 3(d)). The numbers for SBP of both groups were from 155.75 ± 4.62 mmHg (before the second surgery) to 127.39 ± 3.37 mmHg and 145.39 ± 3.47 mmHg (before the second surgery) to 123.16 ± 2.07 mmHg, respectively. Similar trends were seen in DBP. These findings indicated that the increased pressure in Figure 2 was due to the inserted implants, rather than the surgery itself. Therefore, we concluded that the atlantoaxial misalignment/disorder was the main cause for the hypertension in these rats and their blood pressure turned back to normotension when the atlantoaxial disorder was reduced.

### 3.4. Experimentally Caused Hypertension Was Correlated with the Heart Rate and the Blood Acetylcholine Changes

**Figure 4:**
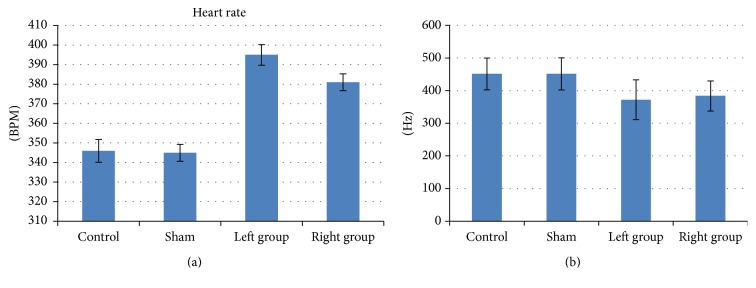
*Hypertension in atlantoaxial disorder rats was correlated with heart rate and blood Ach changes*. On the 7th day of first surgery, the heart rate and the blood Ach level were measured: (a) The heart rate of experimental groups (both LD and RG) was significantly higher than the control and sham groups (*p* < 0.01, either LG or RG compared with the sham group). (b) The blood Ach levels from experimental groups (both LG and RG groups) were significantly lower than those of control and sham groups (*p* < 0.01, either LG or RG compared with the sham group). *n* = 15 for each group. For detailed values see Supplemental Table 2.

Along with the blood pressure increases, heart rates were also enhanced dramatically in both experimental groups on the 7th day after the surgery, and LG had higher beating rates than the RG (Figure 4(a)). Control and sham groups were keeping similar beating rates (Figure 4(a)). The blood acetylcholine (Ach) level was measured from these control and experimental groups (LG and RG) on the 7th day after surgery. The blood ach level measured by enzyme linked immunosorbent assay (ELISA) showed that LG and RG had lower Ach than the control and sham groups (Figure 4(b)). Correlation analysis between experimental groups and the sham group indicates that the high blood pressure is positively correlated with the heart beating rate and negatively correlated with the blood Ach levels, respectively (Table 1 and Supplemental Table 2).

## 4. Discussion

Atlantoaxial disorders are often seen in young adults, but there is no standard diagnosis and treatment in hospital. The atlantoaxial joint is on top of the cervical spine, between the first and second cervical vertebrae. Long-term poor posture may overstretch or constrain surrounding connecting muscle and ligaments, which will cause atlantoaxial disorders. Patients with the disorder are often presenting neck stiffness, pain, activity limitation, more dizziness, blurred vision, and other head and neck syndromes [28, 29]. X-ray imaging is used to diagnose the symptom. In China, general treatments usually consider conservative therapies, including acupuncture [30] and manipulation therapy [31], supplemented by other comprehensive treatment [32]. Hypertension is one of the main symptoms, which is reported with a rate of 44% among these patients [1]. After manipulation therapy to adjust the atlantoaxial joints, most of these patients recovered from hypertension. By taking advantage of animal models, we present here that rats with atlantoaxial disorders were developing hypertension and the high blood pressure turned better when the modeled animals were operated to remove implants that caused the disorder.

The hypertension in our modeled rats is unlikely caused by the stimulation to the vertebral-basilar artery, since early studies had reported that tractions of vertebral artery would barely induce changes of BP [19]. The hypertension may be caused by the stimulation to the superior cervical ganglion (SCG). Activation of SCG would increase the release of noradrenaline (NE) that could contract blood vessels and fasten the heart beating rate (Figure 4), resulting in elevated blood pressure [17, 33]. Decreased level of acetylcholine also happens with the activation of SCG, which is consistent with what we observed in Figure 4. Our method of inserting artificial implants to make atlantoaxial disordered rats is feasible since all successfully modeled animals, assessed by the X-ray imaging system (Figure 1), showed symptoms of hypertension a few days after the surgery. The fact that the LG developed higher blood pressure than RG is consistent with that the asymmetry distribution of the sympathetic nerves on left and right sides [23, 24]. Why the LG has higher blood pressure needs further studies to clarify the function of the asymmetry of sympathetic nerves. The fact that removing the implants lowered the blood pressure further concludes that the disordered atlantoaxial joint is a main cause of hypertension. Taking into consideration that restoration of Atlas alignment is associated with marked and sustained reductions in BP [8], our model highly supports that cervical disorders are associated with hypertension.

We found that in patients with long-term atlantoaxial disorders it was difficult to lower their blood pressure levels by manipulation therapy, while patients being diagnosed shortly will relieve their symptom with a significant effect [15]. This report is consistent with our animal model with their blood pressures being lowered if the disorder was relieved immediately (Figure 3). Our study indicates that these people who may feel head and neck syndromes and have immediate blood pressure increase should see doctors immediately and may receive conservative therapies, such as Chinese medical manipulation.

This study provides experimental basis for the application of Chinese manipulation therapy on atlantoaxial disorders, a phenomenon we observed in our daily practice. Previously we had reported clinically observed cases where hypertension is accompanied with atlantoaxial disorders [34]. We need to further improve our modeling method since inserting implants can only mimic parts of the dislocation of atlantoaxial joints. Besides acetylcholine, more blood hormones such as plasma catecholamine need to be measured. In our future study, we would address whether the hypertension seen in our model rats is caused by the activation of SCG by applying ganglionectomy [35]. Future detailed study on the relationship between the blood pressure and the sympathetic nerves on different sides is required.

## Supplementary Material

Supplemental Figure 1. Process of making the atlantoaxial disorder rat model. Supplemental Figure 2. X-ray imaging of the rats.

## Figures and Tables

**Table 1:** Correlation between blood pressure and the blood Ach.

Parameter	*r*(SBP)/*p* value	*r*(DBP)/*p* value
Ach	−0.32/*p* < 0.01^(1)^	−0.31/*p* < 0.01^(2)^

*Note*. (1) compared with sham group; (2) compared with sham group.

## Abbreviations

Ach: Acetylcholine
BP: Blood pressure
DBP: Diastolic blood pressure
ELISA: Enzyme linked immunosorbent assay
SBP: Systolic blood pressure
SCG: Superior cervical ganglion.
