# Supplementary material for: Atlantoaxial Misalignment Causes High Blood Pressure in Rats: A Novel Hypertension Model
Source: Biomed Res Int. 2017 Jul 16;2017:5986957. doi: 10.1155/2017/5986957 (PMC5534292; doi:10.1155/2017/5986957)
Supplement: Supplementary file 1 — Supplemental Figure 1. Process of making the atlantoaxial disorder rat model. Supplemental Figure 2. X-ray imaging of the rats. [file 5986957.f1.pdf]

Supplemental Figures.

Supplemental Figure 1.Process of making the atlanto-axial disorder rat model.
A. Skin preparation and disinfection. B. Exposure of the atlantoaxial joint with skin incision. C. Preparation of artificial fixtures. D. Implant the fixtures into atlantoaxial joints. E. Sew back the wound. F. Injection with antibiotics.

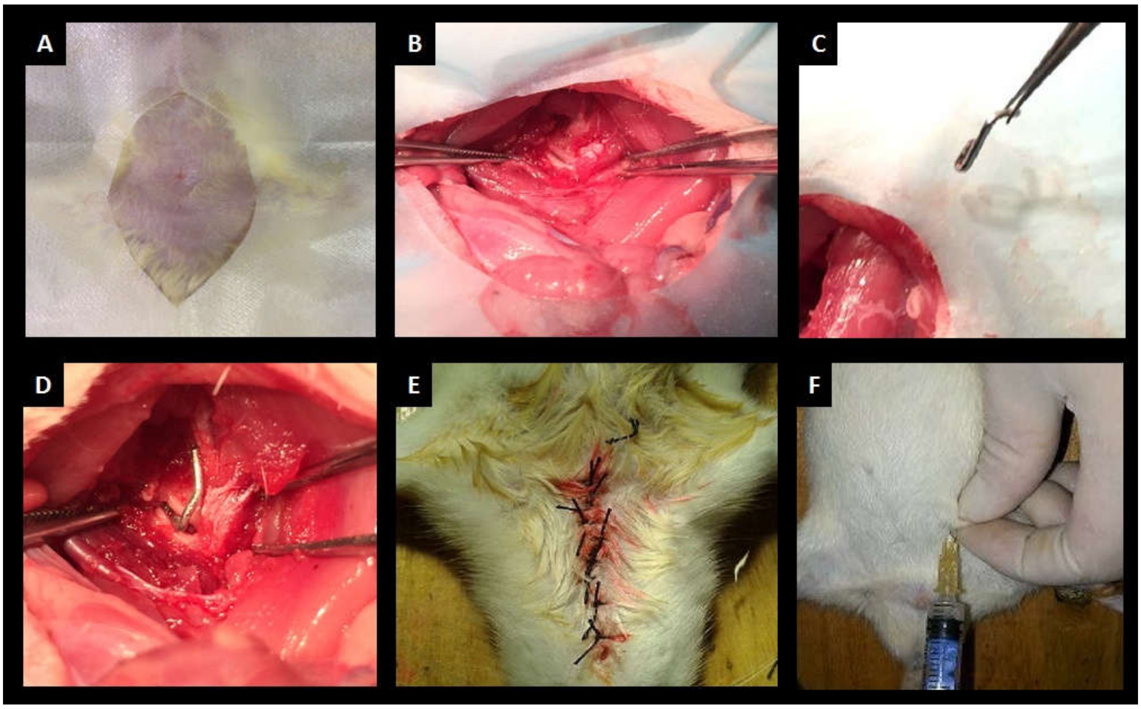

Supplemental Figure 2.X-ray imaging of the rats.

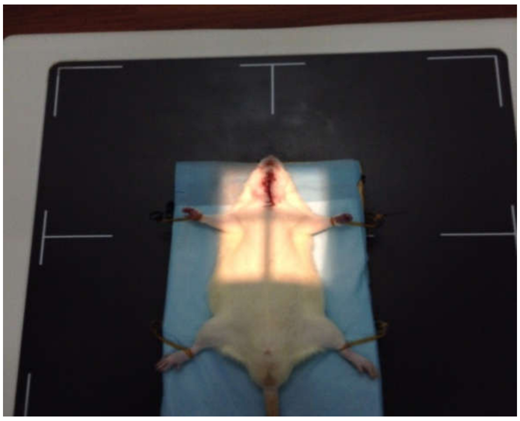

Supplemental Table 1. Blood pressure changes during modeling process.

Date	Blood Pressure	Control Group (M ± se)	Sham Group (M ± se)	Left Group (M ± se)	Right Group (M ± se)
Before Surgery	SBP	122.88±2.82	123.29±2.6	123.11±2.40	123.16±2.07
	DBP	82.52±2.36	82.13±2.64	83.13±1.45	82.56±1.94
1st day after surgery	SBP	122.48±2.99	122.93±2.70	144.00±2.42	126.40±2.67
	DBP	81.86±2.37	82.22±2.47	98.03±2.31	82.96±3.82
2nd day after surgery	SBP	122.66±2.99	123.46±2.70	145.27±3.42	131.40±3.67
	DBP	81.86±2.97	82.22±2.79	131.40±3.67	88.96±3.82
3rd day after surgery	SBP	123.95±1.29	123.47±1.79	149.59±4.73	139.42±5.27
	DBP	83.86±2.13	83.74±2.19	104.82±3.73	95.38±3.89
5th day after surgery	SBP	123.2±1.27	123.98±1.79	155.35±5.82	145.46±4.47
	DBP	81.86±2.73	82.22±2.84	108.39±3.69	100.21±3.59
7thday after surgery	SBP	124.62±2.33	125.01±1.19	155.75±4.62	145.39±3.47
	DBP	83.06±1.37	84.28±1.24	107.19±2.39	103.21±1.59
1st day after removing fixtures	SBP	123.25±2.47	124.79±2.19	135.45±3.92	132.93±4.87
	DBP	82.86±3.13	82.34±3.56	94.88±2.89	87.56±3.97
2nd day after removing fixtures	SBP	124.7±2.76	123.19±2.36	136.45±2.92	128.43±3.17
	DBP	84.36±3.13	83.34±3.56	92.15±2.89	89.76±3.39
5th day after removing fixtures	SBP	123.1±3.47	123.81±2.04	128.69±3.91	126.93±2.14
	DBP	84.31±2.42	83.34±3.26	86.12±3.19	85.76±3.05
7th day after removing fixtures	SBP	124.47±3.39	123.29±2.64	127.39±3.37	125.63±2.54
	DBP	84.58±2.91	83.19±3.26	84.72±3.51	85.96±3.75

Supplemental Table 2.Blood Ach level on 7th day after the surgery.

μg/ML	Control Group	Sham Group	Left Group	Right Group
ACH	506.81±45.71	503.94±48.99[3)]	331.52±57.79[1)]	353.62±55.50[2)]
Heart rate	346±5.81	345±4.29[3)]	395±5.27[1)]	381±4.31[2)]

Note: 1) *p*< 0.01, compared with Sham Group; 2) *p*< 0.01, compared with Sham Group.

3) *p*>0.05, compared with Control Group
